# Retraction Note: Accelerated oral wound healing using a pre-vascularized mucosal cell sheet

**DOI:** 10.1038/s41598-021-82919-5

**Published:** 2021-02-12

**Authors:** Jaewang Lee, Eun Hye Kim, Daiha Shin, Jong-Lyel Roh

**Affiliations:** Department of Otolaryngology, Asan Medical Center, University of Ulsan College of Medicine, Seoul, Republic of Korea

Retraction of: *Scientific Reports* 10.1038/s41598-017-10991-x, published online 06 September 2017

The Editors have retracted this Article.

An institutional investigation found evidence that the images used for Figures 3C, 3D, 3F, 5D and 6E, originated from a different set of experiments to those listed. The Editors therefore no longer have confidence in the integrity of the data presented.

Authors Jaewang Lee and Daiha Shin agree to this retraction. Authors Eun Hye Kim and Jong-Lyel Roh have not responded to any correspondence from the Editors about this retraction.

